# Desiccation Mitigates Heat Stress in the Resurrection Fern, *Pleopeltis polypodioides*

**DOI:** 10.3389/fpls.2020.597731

**Published:** 2020-11-30

**Authors:** Susan P. John, Karl H. Hasenstein

**Affiliations:** Department of Biology, University of Louisiana at Lafayette, Lafayette, LA, United States

**Keywords:** heat stress, hydroperoxide, lipid hydroperoxide, antioxidative enzymes, *Pleopeltis polypodioides*, fatty acids

## Abstract

Although heat and desiccation stresses often coincide, the response to heat especially in desiccation tolerant plants is rarely studied. We subjected hydrated *Pleopeltis polypodioides* fronds to temperatures up to 50°C and dehydrated fronds up to 65°C for 24 h. The effect of heat stress was evaluated using morphological changes, photosystem (PS) II efficiency, and metabolic indicators. Pinnae of dried fronds exposed to more than 40°C curled tighter and became brittle compared to fronds dried at lower temperatures. Exposure to > 50°C leads to discolored fronds after rehydration. Hydrated fronds turned partially brown at > 35°C. Chlorophyll fluorescence (F_t_) and quantum yield (Q_y_) increased following re-hydration but the recovery process after 40°C treatment lasted longer than at lower temperatures. Similarly, hydrated fronds showed reduced Q_y_ when exposed to > 40°C. Dried and hydrated fronds remained metabolically active up to 40°C. Hydroperoxides and lipid hydroperoxides in dried samples remained high up to 50°C, but decreased in hydrated fronds at > 40°C. Catalase (CAT) and glutathione (GSH) oxidizing activities remained high up to 40°C in dehydrated fronds and up to 35°C in hydrated fronds. Major fatty acids detected in both dehydrated and hydrated fronds included palmitic (C16:0) and stearic (C18:0) acids, oleic (18:1), linoleic (C18:2); and linolenic (C18:3) acids. Linolenic acid was most abundant. In dried fronds, all fatty acids decreased at > 35°C. The combined data indicate that the thermotolerance of dry fronds is about 55°C but is at least 10°C lower for hydrated fronds.

## Introduction

Heat stress is one of the major abiotic stresses limiting plant growth, yield, and productivity (Wang et al., 2016). Temperatures beyond the “physiological capacity” of plants have debilitating effects on biochemical processes, cellular homeostasis (Kotak et al., 2007), changes in enzyme activities, photosynthesis (Greer, 2015), protein synthesis (Ferguson et al., 1994), and membrane fluidity (Hemantaranjan et al., 2014). Damage to photosystem (PS) II and associated proteins (Chen et al., 2018) is reported to be a primary target of high temperature stress (Wen et al., 2005; Mathur et al., 2014). Heat stress uncouples electron transport activity, ATP synthesis and variable (F_v_), and maximum (F_m_) chlorophyll fluorescence (Maxwell and Johnson, 2000), which results in the production of reactive oxygen species (ROS) such as singlet oxygen, superoxides, hydrogen peroxide, and hydroxyl radicals, all of which induce oxidative stress (Hasanuzzaman et al., 2013; Awasthi et al., 2015). Furthermore, heat stress affects activity of the antioxidative enzymes superoxide dismutase (SOD), catalase (CAT), peroxidases (POD), ascorbate peroxidase (APX), and low molecular weight antioxidants like ascorbic acid and glutathione (GSH), which change in response to heat stress (Lu et al., 2008; Ergin et al., 2016).

Plants not only experience heat- but also desiccation stress, which stems from loss of water via evapotranspiration. Distinguishing combined stresses is difficult because many of the physiological and molecular responses between these stresses overlap (Zandalinas et al., 2018). Some studies suggest that the combination of these two stresses is more deleterious to plant growth and productivity than each individual stress (Jiang and Huang, 2001; Rizhsky et al., 2002; Lipiec et al., 2013; Zandalinas et al., 2018) and the response to combined stresses cannot be extrapolated from the individual stress (Zandalinas et al., 2018). Therefore, studies typically report the combined effects of heat and drought stress (Rizhsky et al., 2002; Bhardwaj et al., 2015; Zandalinas et al., 2018). However, this approach obscures the individual stress response. Additionally, separating heat from desiccation stress is not possible in the field and difficult to establish even under laboratory conditions but leads to meaningful characterization of the metabolic response to individual stressors.

Plants that are especially well-suited to study desiccation stress are “resurrection” plants. These plants are defined by their vegetative tissues being able to sustain desiccation to water potentials as low as −100 MPa (Gaff, 1997). They can reach complete air dryness and rehydrate without suffering noticeable injury. Because these plants can sustain desiccation and attain certain levels of quiescence during this process, their heat tolerance can be tested in dry and hydrated conditions. A large body of research has reported thermotolerance capacity of non-vascular, poikilohydric species such as bryophytes (Lange, 1955; Nörr, 1974; Meyer and Santarius, 1998) and lichens (Lange, 1953; Tegler and Kershaw, 1981). Although thermo and frost resistance of desiccation tolerant pteridophytes (Kappen, 1964) and heat resistance of desiccation tolerant pteridophytes (Eickmeier, 1986) and angiosperms (Hambler, 1961; Kappen, 1966; Vieweg and Ziegler, 1969) was examined, there is no information on biochemical responses of these plants to elevated temperatures. Based on morphological and photosynthetic responses, there is general consensus that non-vascular and vascular plants show increased thermotolerance after desiccation (Macfarlane and Kershaw, 1978; Tegler and Kershaw, 1981; Eickmeier, 1986). However, the dependency on seasonal conditions also indicates that desiccation alone is insufficient to establish thermotolerance. To dissect the biochemical responses to heat and desiccation, we studied the response of the desiccation tolerant fern *Pleopletis polypodioides* in dry and hydrated fronds. Because pteridophytes are widely distributed and successfully established in various environments, they not only provide essential information on the evolution of desiccation tolerance but also serve an excellent model to study the effects of heat stress.

*Pleopeltis polypodioides* (aka resurrection fern) can tolerate loss of 95% of cellular water content and regain full metabolic activities within a few hours of rehydration (Pessin, 1924; Stuart, 1968; John and Hasenstein, 2017). In response to dehydration, the ventral surface curls inward and the dorsal surface, covered with peltate scales, remains exposed (Pessin, 1924). Such folding mechanisms, also demonstrated by other resurrection plants, are thought to be a defensive mechanism against photooxidation (Farrant et al., 1999; Helseth and Fischer, 2005). Although there is plenty of information on morphological changes of plants in response to heat and desiccation stress, adaptations of ferns to desiccation (Pessin, 1924; Stuart, 1968; Helseth and Fischer, 2005; John and Hasenstein, 2017), and biochemical response to drought (Maslenkova and Homann, 2000; Georgieva and Maslenkova, 2001; Layton et al., 2010; John and Hasenstein, 2018) are limited. In addition, the response of Pleopeltis to heat has not been investigated.

We evaluate the biochemical responses of dry and hydrated fronds of Pleopeltis to heat by assessing their photosynthetic and metabolic activities, levels of stress molecules, activities of antioxidative enzymes, membrane stability, and the fatty acid profile. Our results indicate that independent of hydration, oxidative and antioxidative systems are sensitive to heat but dried fronds tolerate heat better than hydrated fronds.

## Materials and Methods

### Plant Materials

**FIGURE 1:**
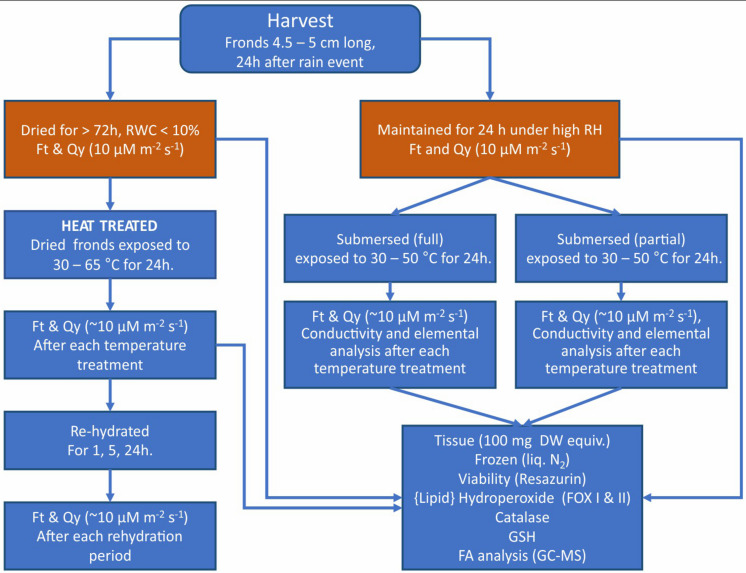
Summary of the workflow of experiments and analyses performed on dried, rehydrated, and submersed Pleopeltis fronds. Data obtained from the initial sample status (red boxes) served a reference for all other treatments.

Because Pleopeltis fronds are highly responsive to environmental conditions and are efficient in water uptake compared to plants with intact rhizomes (John and Hasenstein, 2017), we focused on studying the thermal tolerance of isolated fronds. Hydrated fronds *P. polypodioides* were collected from live oak (*Quercus virginiana*) trees on the campus of the University of Louisiana at Lafayette (30.21 N, −92.02 W). All fronds were collected after a rain event during March to May from 2016 to 2019 and prior to experimentation maintained in a humid chamber at high RH. All fronds were between 4.5 and 5 cm long. The outline of the workflow is illustrated in Figure 1 and individual methodologies explained below.

### Heat Treatment

Heat treatment was applied for 24 h in darkness. Assays of heat treated dry and hydrated (fully and partly submersed) samples were compared with 25°C dry and fresh control, respectively.

#### Dehydrated Fronds

Detached, fresh fronds were dried at 25°C for 72 h, which reduces the RWC to < 5% (John and Hasenstein, 2018). After drying, the fronds were exposed to temperatures between 30 and 50°C for 24 h in an incubator. Relative humidity decreased as a function of temperature increase.

#### Hydrated Fronds

Detached fresh fronds were placed in DI water in 20 mL glass vials such that either the entire frond (vial filled with 20 mL water) or just the apical end (4 mL water) was submersed and exposed to the same temperature as dried fronds. The reason for different levels of submersion was to test the effect of oxygen deprivation in fronds that were fully submersed compared to 100% humidity. The data were compared with hydrated fronds (without immersion) at 25°C.

### Photosynthetic Performance

The photosynthetic performance of the fronds before and after heat stress and after re-hydration was assessed by measuring the minimum chlorophyll fluorescence (F_0_, which is equivalent to F_t_ if the leaf sample is dark-adapted) and quantum yield (Q_y_) using a FluorPen FP100 (Photo Systems Instruments, Czechia). F_0_ was measured by applying a light pulse (455 nm of 0.09 μmoles m^–2^s^–1^ for 30 μs) and recorded as F_t_. Similarly, Q_y_ measurements were obtained by applying a saturating light that measures maximum value of fluorescence (F_m_) from PS II (approximately 3000 μmol m^–2^ s^–1^ for 0.8 s) to dark-adapted dry fronds and dim-light (∼10 μM m^–2^ s^–1^) exposed fronds. Preliminary measurements showed no difference between dark or dim-light adapted measurements. The difference between minimal fluorescence F_0_ and maximal fluorescence F_m_ divided by F_m_, i.e., (F_m_-F_0_)/F_m_. We used this ratio to describe quantum yield (Q_y_) of PS II system. Normal photosynthetic activity shows maximum Q_y_ of about 0.83 (Maxwell and Johnson, 2000; Murchie and Lawson, 2013). We considered reduced values indicative of stress.

The F_t_ and Q_y_ data were collected from fresh, dried and 24 h heat treated fronds as well as after re-hydration for 1, 5, and 24 h at room temperature (25°C). Because obtaining reliable fluorescence measurements from curled (dried) fronds was difficult, the F_t_ and Q_y_ measurements were obtained from flattened fronds that were dried between paper towels and glass plates for 5 days at room temperature. Dried and submersed fronds were examined after temperature treatments. Fronds were rehydrated on water-saturated foam under dim light, and were measured after 1, 5, and 24 h. Q_y_ and F_t_ were identical in dark and dim-light-maintained fronds, we therefore report data of fronds measured under dim light (10 μM m^–2^ s^–1^). The F_t_ and Q_y_ measurements were obtained from the base, center, and the tip of each of six fronds and shown as average of these positions.

### Membrane Permeability

Cell membrane permeability was deduced from the conductivity (μS cm^–1^) of the water in which the fronds were submersed. Conductivity was measured with a MW 301 EC meter (Milwaukee Instruments, Inc.) in 4 mL samples for partially and 20 mL samples in fully submersed fronds and reported as μS normalized to 10 mL. After heat treatments, fronds were kept at room temperature for an additional 24 h to assess re-uptake of ions. Elemental analysis was performed by Inductively Coupled Plasma – Optical Emission Spectrometry (Perkin Elmer, Optima 5300 DV) in 15% HNO_3_.

### Metabolic Activity

In their natural environment, Pleopeltis fronds are exposed to sometimes extreme fluctuations of temperatures, humidity, and light intensities. Our intention is to understand how Pleopeltis responds to heat stress. Because measuring enzyme activities of dried fronds inevitably requires hydration, metabolic activities of dried fronds may not represent the actual activity in the dried state but indicate available metabolites and metabolic capacity at the endpoint of dehydration. Despite these limitations, the following tests were performed with dehydrated and hydrated fronds.

#### Tissue Viability

The reduction of resazurin as described in John and Hasenstein (2017) served as proxy for the assessment of vitality. Pinnae [100 mg dry weight equivalents, calculated as FW/(1-RWC) as documented in John and Hasenstein (2018)] were macerated in 2 mL of 10 mM potassium phosphate buffer (pH 7.2) and 0.2 mL of 4 mM resazurin (Sigma R7017), and incubated under continuous illumination (∼300 μM m^–2^ s^–1^) at 25°C for 3 h before the fluorescence of the supernatant was determined (Varian Cary, λex = 560 nm; λem = 590 nm).

#### Hydroperoxide Determination

Hydroperoxide (and other water-soluble peroxide) content was measured using the ferrous ammonium sulfate/xylenol orange, FOX I assay (Wolff, 1994; Cheeseman, 2006) at 560 nm (John and Hasenstein, 2018). Hydroperoxide quantification in μM was based on a standard curve and calculated as (OD_560_ – 0.004)/0.0763.

#### Measurement of Lipid Peroxide

The FOX II assay was used to quantify lipid peroxides, LOOHs (Jiang et al., 1992; Gay and Gebicki, 2003) according to John and Hasenstein (2018) such that (OD_560_ − 0.1305)/0.0383, determines the BHT equivalent in nM.

#### Catalase Activity

Catalase activity was determined from the decrease of OD_240_ of H_2_O_2_ for 180 s (John and Hasenstein, 2018). Based on 100 mg samples, enzyme activity was expressed as μM × min^–1^ × mg protein^–1^.

#### Consumption of Reduced Glutathione

The consumption of GSH was measured as described earlier (John and Hasenstein, 2018). The assay was modified after (Starlin and Gopalakrishnan, 2013). Samples (100 mg DW equivalent) were extracted in chilled sodium phosphate buffer (40 mM, pH 7, 2 mL) and centrifuged (23,000 *g*, 12 min, 4°C). 500 μL supernatant was transferred into a fresh centrifuge tube, mixed with 400 μL 40 mM sodium phosphate buffer pH 7.0, 100 μL 10 mM sodium azide (Sigma, S-2002), 200 μL DI water, and vortexed. After adding 200 μL (4 mM) GSH (Cayman chemical company, 10007461) and 100 μL 2.5 mM H_2_O_2_, the solution was mixed and incubated for 1 min. The reaction was terminated by adding 500 μL of 10% trichloroacetic acid (Aldrich Chemical Company, 76-03-9). The solution was incubated at RT for 30 min and centrifuged (23,000 *g*, 5 min, 4°C). The supernatant (500 μL) was mixed with 1.5 mL 0.1 M sodium phosphate buffer (pH 7.2) and 1 mL 5, 5’-dithiobis-2-nitrobenzoic acid (Sigma, D8130). After inversion, OD 409 was determined. Enzyme activity is reported as μM GSH consumed min^–1^ × mg protein^–1^.

### Protein Determination

Protein content for the enzyme assays was determined according to Bradford (1976) using ovalbumin (Sigma, A-5378) as standard.

### Fatty Acid Analysis

Fatty acids were extracted from tissue equivalent to 100 mg fresh weight, derivatized to their methyl esters, and quantified (John and Hasenstein, 2018) using an Agilent (Wilmington, DE, United States) gas chromatograph (6890) with mass selective detector (5973). One μL samples were injected and separated on a Phenomenex Zebron ZB-5MS column with hydrogen as carrier gas at a flow rate of 1.8 mL min^–1^. The temperature profile started at 100°C for 2 min, ramped (10°C × min^–1^) to 280°C and held for another 8 min. Identification of compounds was based on the National Institute of Science and Technology library 2008 in Chemstation software (E.02.02.1431). The quantity of individual fatty acids was determined based on retention time and slope of derivatized palmitic acid (Sigma, P-5917; Rt = 11.7 min; 3.98 × 106 TIC ng^–1^), linoleic acid (Sigma, L1376; Rt = 13.3 min, 3.53 × 106 TIC ng^–1^), linolenic acid (Cayman Chemical Company, 90210; Rt = 13.37 min; 2.51 × 106 TIC ng^–1^), oleic acid (Sigma, O1630; Rt = 13.4 min; 1.88 × 106 TIC ng^–1^), and stearic acid (Sigma, S-4751; Rt = 13.6 min; 3.05 × 106 TIC ng^–1^). Tissue samples were analyzed under identical conditions as standards.

### Statistical Analysis

The data are reported as means of three to six biological replicates obtained from individual fronds. Analysis of variance (ANOVA) with Tukey-Kramer *post hoc* test was performed using Excel data analysis tool pack (Microsoft Corporation, 2016).

## Results

### Morphological Response

**FIGURE 2:**
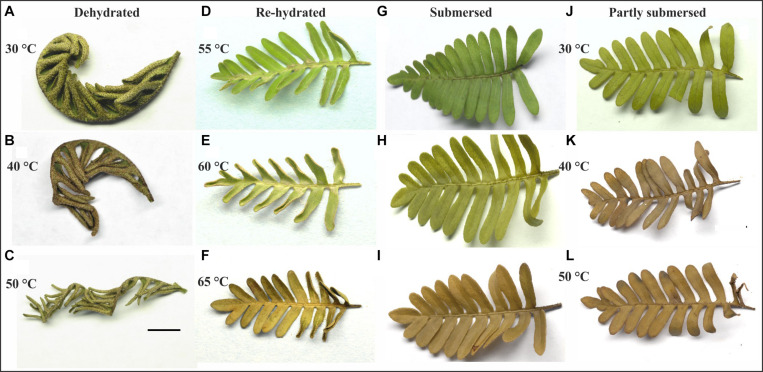
Response of dehydrated **(A,B,C)**, submersed **(G,H,I)**, and partly submersed **(J,K,L)** fronds to 24 h heating at 30, 40, and 50°C, and fronds re-hydrated after exposure to 55, 60, and 65°C **(D,E,F)**. Exposure to ≥ 40°C resulted in tight curling of dehydrated fronds and browning of the hydrated fronds. Re-hydrated fronds show discoloration near the rachis between 55 and 60°C and complete discoloration of the frond after 65°C. Scale bar = 1 cm.

Pinnae of dried Pleopeltis fronds curled tighter with increasing temperature treatment (Figures 2A–C). Dried tissue showed no effects, but discolorations became visible after rehydration. Exposure to 55°C resulted in patchy browning along the mid-rib (rachis) of the frond (Figure 2D); after 60°C fronds were slightly (Figure 2E) and after 65°C completely discolored (Figure 2F). Submersed (Figure 2G) and partly submersed (Figure 2J) fronds showed no effects at 30°C but after exposure > 40°C submersed (Figures 2H,I) and partly submersed (Figures 2K,L) fronds showed browning.

### Photosynthetic Performance

#### Dried Frond

**FIGURE 3:**
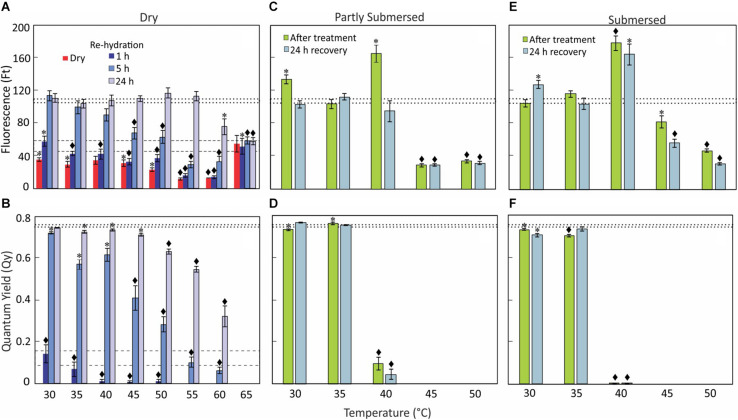
Chlorophyll fluorescence, F_t_ **(A,C,E)** and Q_y_ **(B,D,F)** data of dry, re-hydrated, partially, and fully submersed Pleopeltis fronds after 24 h exposure to different temperatures. Control values ± SE @ 25°C for F_t_ and Q_y_ of dry fronds are indicated by dashed lines **(A,B)** and for rehydrated fronds by dotted lines **(A–F)**. The F_t_ and Q_y_ of heat-treated, dry samples are compared with 25°C dry samples **(A,B)**; dried fronds did not show measurable Q_y_ values regardless of temperature. Re-hydrated and hydrated samples are compared with 25°C fresh samples **(C–F)**. Significant differences between controls and treatments are indicated by * (*P* ≤ 0.05) and ◆ (*P* ≤ 0.001); means ± SE, *n* = 6.

Instantaneous fluorescence (F_t_) of treated dried fronds measured lower than (control) dry fronds at 25°C (Figure 3A) while Q_y_ of fronds exposed to 30°C and higher was not detectable (Figure 3B). Interestingly, F_t_ of 65°C treated samples was higher than 60°C and not different from control (Figure 3A), whereas Q_y_ even after rehydration remained undetectable (Figure 3B).

#### Re-hydrated Fronds

After rehydration, the chlorophyll fluorescence (F_t_) increased over time and reached control levels within 5 h for temperatures up to 35°C (Figure 3A). However, exposure to higher temperatures (≤55°C) required a recovery period of 24 h. Despite the high F_t_ values, the Q_y_ decreased with higher temperatures (Figure 3B). F_t_ of 1-h re-hydrated fronds was low compared to (fresh) control but measurable at all temperature ranges, i.e., 30–65°C (Figure 3A). However, the Q_y_ value decreased from 30 to 50°C and was not detectable at > 50°C (Figure 3B). Similarly, F_t_ of fronds re-hydrated for 5 h after less than 40°C showed values similar to (fresh) controls (Figure 3A) but F_t_ was lower in rehydrated fronds exposed to ≥ 45° [*F*(5,30) = 18, *P* < 0.0001]. The Q_y_ of 5 h re-hydrated fronds was lower than in controls [*F*(8,45) = 37.5, *P* < 0.0001] but greater than after 1 h re-hydration [*F*(1,94) = 55.8, *P* < 0.0001] (Figure 3B). Beyond 45°C, Q_y_ efficiency decreased steadily with increasing temperature (Figure 3B); there was no recovery after exposure to 65°C. Based on these data, Q_y_ is a better indicator of sensing heat than F_t_.

#### Hydrated Fronds

Despite some increase after 30 and 40°C treatments, F_t_ of partially submersed fronds decreased after exposure to temperatures higher than 40°C [*F*(2,15) = 312, *P* < 0.0001] (Figure 3C); in contrast Q_y_ was already significantly reduced at 40°C [*F*(1,10) = 217, *P* < 0.0001] (Figure 3D). After recovery (at room temperature for 24 h), F_t_ and Q_y_ of 30 and 35°C treated samples did not differ from controls (Figures 3C,D). However, in 40°C treated samples Q_y_ remained significantly lower than base-line values [*F*(1,10) = 711.6, *P* < 0.0001], which suggests that hydration is beneficial for samples exposed to temperatures below 40°C. Unchanging but measurable F_t_ values in ≥ 45°C fronds and the lack of recovery of Q_y_ after 40°C indicates degradation of PS II.

In fully submersed samples, chlorophyll fluorescence was higher but Q_y_ suppression was stronger than in partly submersed fronds (Figure 3F). All heat-stressed samples showed reduced F_t_ values after the recovery period, indicating that chlorophyll degradation continues during this time. The Q_y_ values of ≥ 40°C treated samples remained undetectable (Figure 3F). Overall, the samples exposed to ≥ 40°C showed a response similar to partially submersed fronds. The data clearly show that hydration renders the photosynthetic apparatus more sensitive to heat stress than dried fronds. Importantly, the similarity between partial and full hydration suggests that reduced gas exchange in submersed fronds is not the cause of the decline of Q_y_.

### Ion Leakage

**FIGURE 4:**
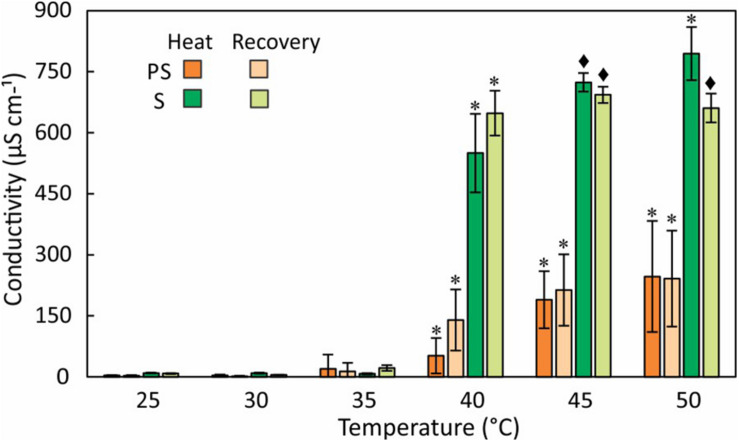
Electrolyte leakage measured as conductivity of external water after 24 h at various temperatures and after a 24 h recovery period at RT. Differences in conductivity between heat treated and 25°C control are indicated by * (*P* ≤ 0.05) and ◆ (*P* ≤ 0.001); means ± SE, *n* = 6.

To assess the response of cell membranes to heat stress, we measured the conductivity of external water from partly and fully submersed samples treated between 30 and 50°C (Figure 4). Although the fronds were washed before the experiments to minimize surface contamination, the conductivity (normalized to 10 mL) at 30 and 35°C were not different from controls, did not differ between partly and fully submersed samples, and likely represents surface-bound ions or normal ion exchange. However, treatment of ≥ 40°C strongly increased the conductivity of fully and partly submersed fronds, indicating membrane leakiness (Figure 4). There was no reduction in conductivity after 24 h recovery at RT (Figure 4); thus, there was no re-uptake of ions during the recovery phase. The conductivities of submersed samples were higher than those of partly submersed samples (Figure 4). The ion concentration was clearly dependent on the extent of immersion, indicating that long-distance transport did not contribute to the ion release.

**FIGURE 5:**
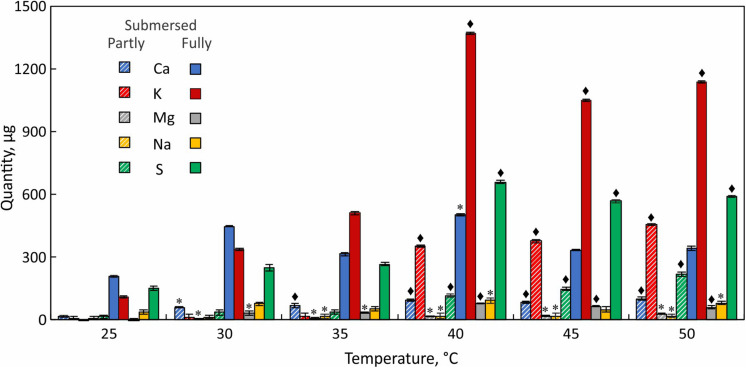
Elemental analysis of leachate from partially (hatched bars) and fully submersed (solid bars) Pleopeltis fronds. Significant differences between heat treated and 25°C control are indicated by * (*P* ≤ 0.05) and ◆ (*P* ≤ 0.001); means ± SE, *n* = 3.

Elemental analysis of the leachate revealed that K^+^ is the major released ion followed by Ca^+2^ and S (Figure 5). Na^+^ and Mg^2+^ were present at lower quantities. K^+^ and S in the leachate increased with temperature and both elements showed the largest increase at 40°C (Figure 5). Ca^+2^ loss occurred more evenly across the tested temperature range and like Na^+^ did not show a distinct threshold temperature (Figure 5). Unlike partly submersed samples, the ions from submersed fronds did not increase beyond 40°C, which indicates that membrane damage occurs at that temperature.

### Reducing Capacity

**FIGURE 6:**
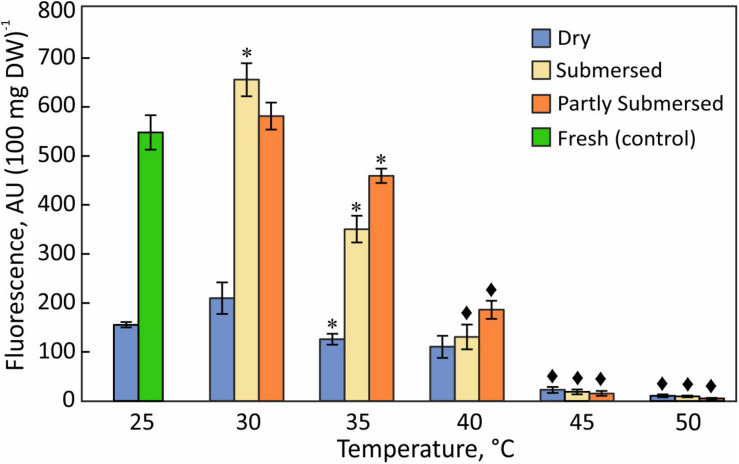
Reduction of resazurin in dried fronds and hydrated (fully and partly submersed) fronds exposed to heat for 24 h. The metabolic activity of heat treated dry and hydrated fronds is compared with 25°C dry and fresh (control), respectively. Significant differences between treated and controls are indicated by * (*P* ≤ 0.05) and ◆ (*P* ≤ 0.001); means ± SE, *n* = 6.

Generic reducing capacity represents overall metabolic activity. Dried fronds showed significantly lower activity than hydrated tissue [*F*(4,25) = 19.6, *P* < 0.0001] below 45°C but their reducing capacity exceeded that of submersed tissue at higher temperatures (Figure 6) regardless of the type of submersion. Despite declining reducing capacity of dried fronds at elevated temperatures, dried fronds showed metabolic activity. Likewise, the reducing capacity remained higher in partly than in fully submersed fronds [*F*(1,10) = 4.9, *P* = 0.05] (Figure 6).

### Hydroperoxides

**FIGURE 7:**
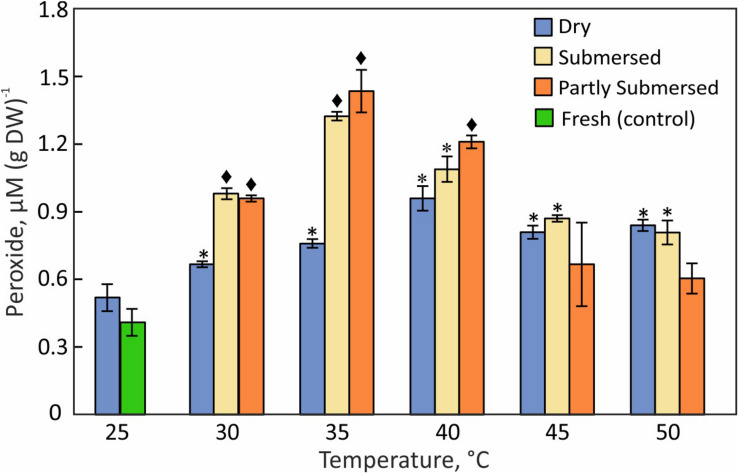
Hydroperoxide content in dried, fully, or partly submersed Pleopeltis fronds exposed to various temperatures for 24 h. The content from heat treated dry and hydrated fronds is compared with 25°C dry and fresh (control), respectively. Significant differences between treated and controls are indicated by * (*P* ≤ 0.05) and ◆ (*P* ≤ 0.001); means ± SE, *n* = 4.

The hydroperoxide content of dried fronds increased gradually with temperature and reached a maximum at 40°C (Figure 7). Fully and partly submersed fronds responded similarly and showed their highest values at 35°C (Figure 7). Higher temperatures led to a decrease on peroxides that was less in dried than in submersed fronds. The moderate increase in dry but stronger increase in hydrated fronds suggests that peroxide accumulates during heat stress but declines when the stress exceeds the metabolic capacity.

### Lipid Hydroperoxides

**FIGURE 8:**
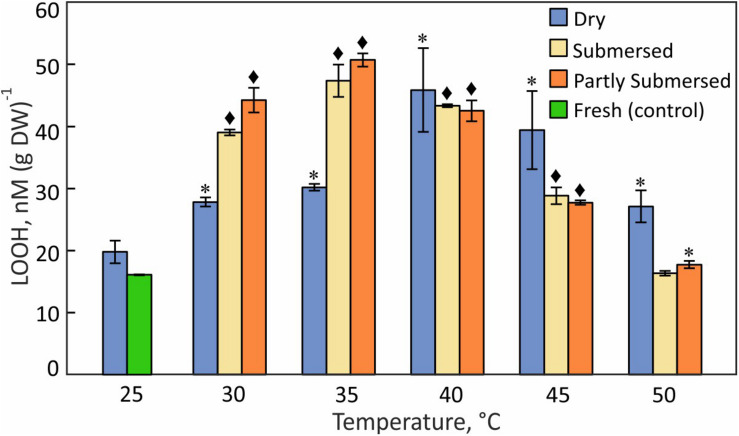
Lipid hydroperoxide (LOOH) content in dried and fully or partly submersed Pleopeltis fronds exposed to different temperatures for 24 h. The content from heat treated dry and hydrated fronds is compared with 25°C dry and fresh (control), respectively. Significant differences between treated and controls are indicated by * (*P* ≤ 0.05) and ◆ (*P* ≤ 0.001); means ± SE, *n* = 4.

The LOOH pattern was similar to hydroperoxides; dried fronds showed a steady increase with temperature with a maximum at 40°C [*F*(4,15) = 3.7, *P* = 0.03] (Figure 8). Partly and fully submersed samples reached their maximum at 35°C but showed lower levels of LOOH than dried fronds at 45 and 50°C (Figure 8), likely related to damaged metabolism, which weakens the response. Therefore, similar to the hydroperoxide profile, the maximum LOOH values signal the onset of non-compensated, i.e., damaging, heat stress.

### Catalase Activity

**FIGURE 9:**
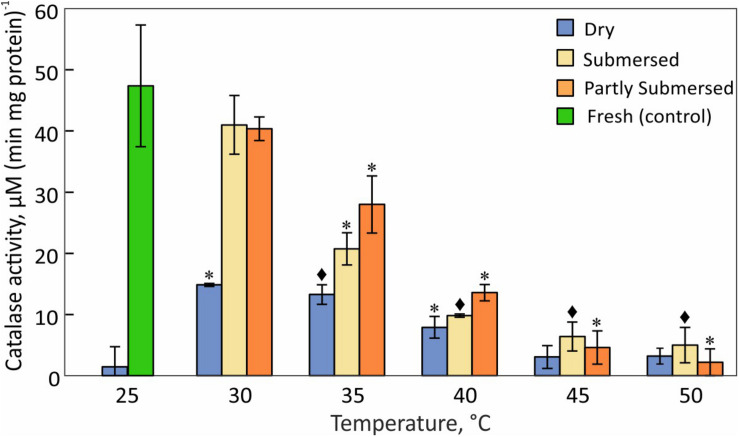
Catalase activity in dried and fully or partly submersed Pleopeltis fronds exposed to different temperatures for 24 h. The activity of heat treated dry and hydrated fronds is compared with 25°C dry and fresh (control), respectively. Significant differences between treated and controls are indicated by * (*P* ≤ 0.05) and ◆ (*P* ≤ 0.001); means ± SE, *n* = 4.

The greatest difference between dried and fresh tissue was measured at 25°C (Figure 9). Dried fronds showed their highest CAT activity at 30°C but the decline at higher temperatures was less than in hydrated fronds [*F*(2,57) = 3.1, *P* = 0.05]. Like dry fronds, submersed fronds showed a steady decline in CAT activity and the extent of submersion had no effect (Figure 9). Because CAT activity in dried fronds was significantly higher after 30°C treatment than at 25°C and significantly reduced in hydrated fronds at 35°C, CAT activity was higher in hydrated vs. dried tissue. CAT activity is remarkably temperature sensitive and the decline at 35°C indicates a stress response of CAT-linked processes.

### Glutathione (GSH) Consumption

**FIGURE 10:**
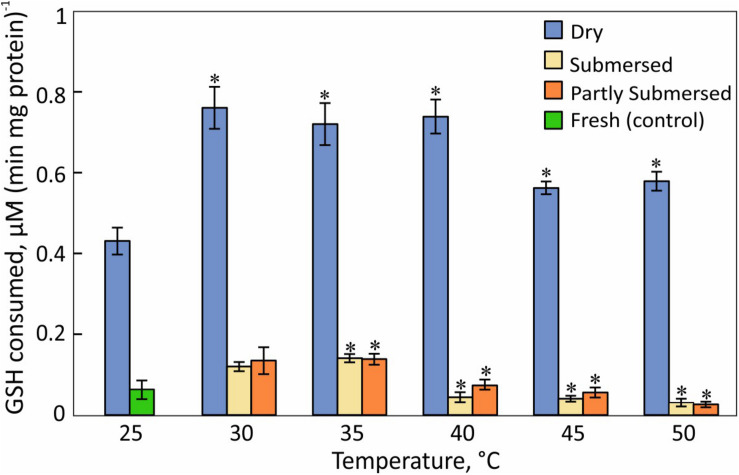
H_2_O_2_-dependent glutathione oxidation activity in dried and partially and fully submersed Pleopeltis fronds exposed to different temperatures for 24 h. The activity of heat treated dry and hydrated fronds is compared with 25°C dry and fresh (control), respectively. Significant differences between treated and controls are indicated by ^∗^ (*P* ≤ 0.05); means ± SE, *n* = 4.

Glutathione consumption responded stronger to desiccation than heat stress [*F*(2,57) = 363, *P* < 0.0001] (Figure 10). GSH consumption in dried fronds decreased at ≥ 45°C [*F*(1,6) = 13.3, *P* = 0.01] compared to 30–40°C treated samples (Figure 10). GSH consumption of hydrated fronds showed a similar reduction and the extent of submersion had no effect. GSH utilization of 30°C dried and hydrated samples did not differ from the respective 25°C controls.

### Fatty Acid (FA) Profile

**FIGURE 11:**
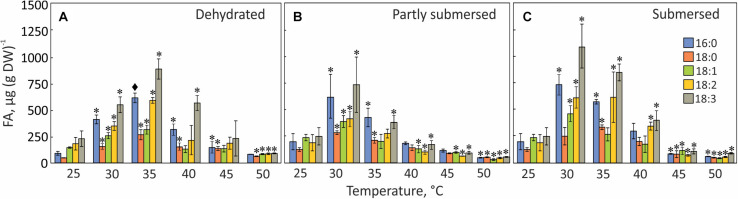
FA profiles in dried **(A)**, partly submersed **(B)**, and fully submersed **(C)** fronds after exposure to various temperatures for 24 h. 16:0 = palmitic acid; 18:0 = stearic acid; 18:1 = oleic acid; 18:2 = linoleic acid; 18:3 = linolenic acid. Significance levels are based on the FA content of 25°C dry and hydrated fronds, respectively. Significant differences between treated and controls are indicated by * (*P* ≤ 0.05) and ◆ (*P* ≤ 0.001); means ± SE, *n* = 4.

Temperature stress changed the FA composition and quantity in all samples. The total FA content in dehydrated fronds increased up to 35°C but decreased at higher temperatures (Figure 11A). In partly and fully submersed fronds, the total fatty acid reached its peak at 30°C and declined at higher temperatures (Figures 11B,C); the FA content in 50°C treated dried and hydrated samples was 76% and 90% lower than 30°C treated fronds, respectively (Figure 11).

Palmitic (16:0), stearic (18:0), oleic (18:1), linoleic (18:2), and linolenic (18:3) acid were detected in controls and heat-treated fronds but linolenic acid (18:3) was the dominant FA at all temperatures and the total amount of fatty acids in fully submersed fronds was higher than in dried and partly submersed samples (Figure 11).

The extent of unsaturation correlated with peroxidation; therefore, we examined the degree of unsaturation for each temperature. The percentage of unsaturated fatty acids (18:1, 18:2, 18:3) in 30°C, dried and partly submersed samples was 12 and 5% lower than at 25°C (Figure 11). Exposure to temperatures higher than 30°C decreased unsaturation, especially in partly submersed samples (Figure 11 and Supplementary Table 1). Regardless of the hydration condition, after exposure to temperatures of 45°C and higher, all FAs showed reduced but equal quantities. In general, the relative proportion of unsaturated fatty acids was higher in dried than in hydrated fronds; and declined in dried fronds above 40°C; this reduction began in hydrated fronds at 35°C.

## Discussion

Our data describe diverse responses to separate desiccation and high temperature stresses. As stated under section “Materials and Methods,” it is important to reiterate the arbitrary nature of the study. The problematic aspects include the duration of the temperature treatment and the testing of various processes and enzymatic activities. It is unlikely that in nature temperatures greater than 40°C extend for more than 12 h. Nonetheless, heat tolerance was tested previously for an equally arbitrary time of 30 min, which resulted in tolerance of > 100°C (Lange, 1955). While evaporative cooling likely provides some protection during short-term heat exposure, our heat treatments of 24 h exceeds naturally occurring conditions but provides enough time to assess metabolic responses. Similarly, submersion of fronds eliminates water stress but may cause different stress for the epiphytic Pleopletis as was shown by the lower reducing capacity (Figure 6) and reduced Q_y_ (Figure 3F) of completely vs. partially submersed fronds (Figure 3D). This sensitivity may be the result of reduced gas exchange (Voesenek et al., 2006), or the enhanced activity of symbiotic surface organisms (Koch and Barthlott, 2009); both phenomena can lead to anoxia (Blokhina et al., 2003; Banti et al., 2010). Nonetheless, the differences between the submersion treatments illustrate the sensitivity of the fronds to different conditions and allow for nuanced evaluation of heat effects.

Stress response includes changes in morphology, cellular functions, metabolism, and ultimately gene expression. Because of the reduced metabolism under dry conditions, it is likely that dry tissue shows reduced susceptibility to heat damage. The co-variance between desiccation and heat may induce changes in cell wall and thus frond shape (Wu et al., 2018) a suitable indicator for heat stress of dry material (Figure 2). However, fronds expanded even after exposure to 65°C; therefore, heat stress cannot be assessed solely through changes in shape or color.

### Changes in Frond Shape and Color

In response to increasing temperature, dehydrated Pleopeltis fronds became thinner, pinnae curled tighter (Figures 2B,C), and after exposure to ≥ 45°C fronds became brittle. Such dramatic changes are likely a response to the combination of water loss and heat that reduces matrix hydration and therefore affects shape and elasticity of cell wall polymers (Lima et al., 2013). These changes possibly indicate glass transition, which facilitates survival in ferns spores (Ballesteros et al., 2017). Among the cell wall polymers, pectin affects the elasticity of the cell wall (Willats et al., 2001) and heat sensitivity (Lima et al., 2013). Loss of pectin functionality leads to increased cell wall porosity, brittleness, and conductive evaporation (Willats et al., 2001; Bethke et al., 2016). Pectin arrangements are likely modified in response to the combination of drying and elevated temperatures, but further research is needed to assess the status of pectin in the cell wall of dried Pleopeltis fronds. Fronds exposed to 30–50°C fully expanded upon rehydration (at 25°C) and did not show necrosis, or changes in shape of the fronds (data not shown).

Discolorations of hydrated fronds after exposure to ≥ 35°C (Figures 2E–I) and re-hydrated fronds after exposure to > 55°C (Figure 2) indicate that these fronds have experienced stress beyond their thermal tolerance resulting in chloroplast or chlorophyll degradation, which eventually leads to senescence (Jespersen et al., 2016) and programmed cell death (PCD) (Qu et al., 2009; Wang et al., 2015). One of the key enzymes known to regulate the breakdown of chlorophyll is pheophytinase (Schelbert et al., 2009). It is possible that the activity of this enzyme increases at high temperatures, similar to observations in bentgrass (Jespersen et al., 2016). Stress-induced chlorophyll degradation is also affected by ethylene, abscisic acid, cytokinin, and ROS (Xu and Huang, 2007). These compounds also influence PS II-mediated electron transport (Xu and Huang, 2007; Griffiths et al., 2014), and membrane-bound ion channels (Demidchik, 2018), proteins, and lipids (Veerasamy et al., 2007; Griffiths et al., 2014). Based on browning, our morphological data indicate that 40°C is the threshold for heat damage for hydrated Pleopeltis fronds (Figures 2E–I); in contrast, dried fronds can tolerate > 60°C (Figure 2).

### Photosynthetic Susceptibility

#### Dried Fronds

Fluorescence (F_t_) and quantum yield (Q_y_) of dried fronds were lower than in fresh fronds but readily detectable (Figures 3A,B), indicating that drying decreases but not completely blocks photosynthetic activity. After dry fronds were exposed to > 50°C, Q_y_ was undetectable (Figure 3B) and F_t_ decreased (Figure 3A) suggesting that the PS II system responds to stress and is blocked by heat (Yamada et al., 1996). This disruption likely protects the photosynthetic apparatus from oxidative damage. Interestingly, after exposure to 65°C, F_t_ was higher than after 60°C exposure or in non-heat-treated, dry fronds (Figure 3A), suggesting that extreme temperatures (i.e., 65°C) lead to the disintegration of the photosynthetic reaction center (Wise et al., 2004; Kadir et al., 2007) but chlorophyll fluorescence persists. Thus, based on the F_t_ and Q_y_ data, the photosynthetic apparatus in dry fronds is heat-tolerant up to 60°C. The photosynthetic apparatus adjusts to stress in several ways, including elevated non-photochemical quenching (NPQ), which can lead to increases in Zeaxanthin and other pigments even under dark conditions (Brüggemann et al., 2009; Leuenberger et al., 2017) and ROS (Ramel et al., 2012). Thus, pigment metabolism may be an important element of stress response.

The recovery of the photosynthetic performances (F_t_ and Q_y_) following re-hydration was time dependent. Q_y_ values of fronds exposed to 30°C reached control values within 5 h of re-hydration but required at least 24 h in fronds exposed to higher temperatures (Figure 3B). The slow recovery of Q_y_ performance indicates that heat exerts long-term stress on the PS II system, likely by interfering with the water oxidation complex (Srivastava et al., 1997) and/or PS II energy transfer (Kadir et al., 2007). Fronds re-hydrated after 60°C appeared photosynthetically functional but at significantly reduced efficiency (Figures 3A,B), which suggests that repair processes are damaged (Allakhverdiev et al., 2008). Photosynthetic activity (Q_y_) in re-hydrated fronds after 65°C was not detectable (Figure 3B), and indicates irreversible damage (Kadir et al., 2007). Thus, similar to morphological data, F_t_ and Q_y_ measurements indicate that the maximal heat tolerance of dehydrated Pleopeltis is 60°C. These data indicate that chlorophyll and/or the reaction center remain intact in spite of absence of PS II activity. The lack of photosynthetic activity may protect the photosynthetic apparatus from damage.

#### Hydrated Fronds

F_t_ and Q_y_ of partly and fully submersed (Figures 3C–F) fronds exposed to ≤ 35°C were not affected. However, at 40°C, Qy decreased while F_t_ increased. Similar to observations in dried tissue, this unequal response indicates a dichotomy between chlorophyll stability and reduced photosynthetic efficiency, possibly as a result from inactivation of the oxygen evolving system (Wise et al., 2004) and/or the PS II reaction center (Bolharnordenkampe et al., 1989). The reduction of F_t_ at > 40°C and non-detectable Q_y_ (Figures 3D,F) indicates that the fronds become photosynthetically non-functional beyond 40°C and identifies the thermal tolerance of hydrated fronds as 40°C.

### Electrolyte Leakage

An increase in conductivity of the external medium indicates cell membrane damage and efflux of ions (Ilik et al., 2018). High conductivity in fully submersed samples relative to partly submersed suggests that the integrity of the membrane is affected by the combination of heat and extent of immersion. Further, the absence of gas exchange seems to negatively impact membrane stability, especially at temperatures > 35°C. Low conductivity in partly and fully submersed samples at ≤ 35°C and no difference from the control likely represents regular ion exchange in intact membranes that is maintained until about 35°C (Figure 4A). During the ensuing 24 h recovery period, the (slight) decrease in conductivity suggests at least partial reuptake of ions (Figure 4B). Since re-absorption is an active process, it is likely that this process is dependent on the activity of plasma membrane proton ATPase, which is reported to be activated by the combination of high temperature and resulting membrane lipid modification (Viegas et al., 1995).

Leaching from leaves is broadly defined as removal of substances from plant leaves by action of rain, fog, dew, and washing (Tukey, 1970). Other factors such as stress, water content, nutrient availability, and physical characteristics of the tissue also influence the extent of leaching (Tukey, 1970). In partly submersed samples, presence of relatively lower amount of water and interaction of vapor mixture with the tissue surface may contribute to leaching. Additionally, since Pleopeltis fronds readily absorb water (John and Hasenstein, 2017), it is likely that water permeability contributes to ion loss. The higher quantity of ions in the leachate (Figure 5) and high conductivity (Figure 4) in fully submersed samples compared to partly submersed samples is likely proportional to the extent a submersion but indicates the deleterious effect of heat on membrane stability. The most abundant ion found in the leachate was K^+^ (Figure 5) consistent with its vital role in the osmoregulation (Cochrane and Cochrane, 2009). Loss of ions in general likely triggers ROS production that leads to oxidative stress and eventually cell death (Demidchik et al., 2014). Taken together, photosynthetic data (Figure 3) and cation leakage (Figure 4) suggest that thermal tolerance of hydrated fronds is 40°C.

### Metabolism

Metabolically active cells rely on a steady supply of reduced compounds; therefore, the concentration of reduction equivalents is a measure of metabolic health. However, assessing the metabolic status of dried Pleopeltis through aqueous redox dyes is problematic because tissue rehydrates in the dye solution. Therefore, our data report the reduction capacity of dried fronds immediately after hydration. Nonetheless, the temperature and hydration variability of the data indicates that the measurements provide a meaningful estimate of the reduction capacity. The declining reducing capacity with increasing temperatures (Figure 6) is likely the result of diminished electron transport (Hüve et al., 2011)and uncoupled photosynthetic electron transport (Sharkey and Zhang, 2010; Awasthi et al., 2015). Because metabolic activity of dried fronds was detectable, dehydrated Pleopeltis remain metabolically active up to at 40°C (Figure 6). Metabolic changes in desiccated moss tissue resulted in a glassy state only after rapid desiccation where enzymatic reactions were absent. In contrast, slow drying achieved a “rubbery state” (Fernandez-Marin et al., 2013). We assume that the slow drying of Pleopeltis and its maintained flexibility is indicative of a non-glassy state with reduced metabolism.

Hydrated fronds showed higher metabolic activity than dried fronds up to 40°C (Figure 6) but similar values at higher temperatures. This observation corresponds to the heat sensitivity of imbibed seeds, which deteriorate at high temperature compared to dried seeds (Hill and Johnstone, 1985). The metabolism of hydrated fronds is similar to the photosynthetic performance (Figures 3C–F) and is similar to the thermotolerance of other tropical and sub-tropical plants (Hemantaranjan et al., 2014). Exposure of plants to heat loads beyond their tolerance level leads to senescence, necrotic symptoms (Figures 2E–I), or apoptosis (Reape and McCabe, 2008). Apoptosis or PCD is typically estimated by the loss of metabolic functions (as in Figure 6) and characterized by cell shrinkage, nuclear condensation and fragmentation, and breakup of a cell into small apoptotic bodies (van Doorn et al., 2011). In contrast, necrosis is characterized as a chaotic and uncontrolled form of cell death that involves early rupture of the plasma membrane (van Doorn et al., 2011) and confined swelling of the cell (Reape et al., 2008). Viability stains can assess overall cell health but cannot distinguish between PCD and necrosis (Reape et al., 2008). To determine the type of cell death that occurs in Pleopeltis in response to heat stress, examination of endomembranes, nuclease activity, and DNA integrity would be essential, as has been shown in carrot (McCabe et al., 1997) and tobacco cells (Burbridge et al., 2006).

### Reactive Oxygen Species and Antioxidant Enzyme Activities

The increase of ROS (Figure 7) in dry and hydrated fronds suggests that Pleopeltis experiences oxidative stress (Ali et al., 2005; Awasthi et al., 2015). The decline of ROS for hydrated but not dried fronds at > 40°C indicates that dehydrated fronds are capable of coping with elevated ROS at higher temperatures than hydrated fronds. The high levels of peroxides at 40°C (Figures 7, 8) also support heat tolerance to about 40°C.

Pleopeltis, like most plants, responds to heat by enhanced production of ROS (Hemantaranjan et al., 2014), which in turn activate signal transduction and ultimately defense mechanisms and PCD (Burbridge et al., 2006). Therefore, the decrease of hydroperoxide (Figure 7) and lipid hydroperoxide (Figure 8) above 40°C indicates damaging, possibly lethal, shifts in the redox equilibrium (Foyer and Noctor, 2005; Suzuki et al., 2012).

In addition to elevated ROS, the decreased availability of antioxidants or activity of reducing enzymes likely enhances ROS toxicity. CAT, an important enzyme for the detoxification of H_2_O_2_, is sensitive to elevated temperatures (Figure 9) and has been reported to decline upon dehydration (John and Hasenstein, 2018). The heat correlated reduction of CAT activity suggests that CAT is unable to detoxify ROS as also reported by Ali et al. (2005). Reduced CAT activity and high amounts of hydroperoxides may lead to an oxidative environment that then enhances consumption of GSH, a phenomenon also observed in Arabidopsis (Queval et al., 2011) and barley mutants (Smith et al., 1985) that lack CAT activity. GSH-oxidation increases during dehydration (John and Hasenstein, 2018) and heat-stressed, dried fronds maintain their high GSH levels up to 40°C (Figure 10). Thus, GSH oxidizing enzymes have greater heat tolerance than CAT. It is also possible that the accumulation of the oxidized form of GSH (GSSG) in chloroplasts and vacuoles (Queval et al., 2011) is important for redox regulation, especially during stress (Smith et al., 1985; Tommasini et al., 1993).

Hydrated fronds accumulate high levels of hydro- (Figure 7) and lipid hydroperoxide (Figure 8) up to 40°C. These stress indicators could be a response to electrolyte leakage (Figure 4), as reported for bentgrass (Liu and Huang, 2000). However, unlike bentgrass which showed increased lipid peroxide and electrolyte leakage, Pleopeltis exhibited increased electrolyte leakage (Figure 4) but LOOH content decreased at > 40°C (Figure 8), which could result from interactions between peroxide (Figure 7) and membrane-bound unsaturated fatty acids (Figures 11B,C) (Blokhina et al., 2003). These results suggest that (hydrated) Pleopeltis membranes are affected even by temperatures below ≤ 40°C. Because CAT (Figure 9) and GSH consuming enzymes (Figure 10) may protect membranes, further research is needed to determine temperature effects on membrane integrity.

### Fatty Acids

Stress-responsive metabolites in plants include fatty acids (Su et al., 2009; Xu et al., 2010; Zhong et al., 2011; Ahmad et al., 2013). Our data confirm this notion because the FA composition of *Pleopeltis* fronds changes with temperature and hydration (Figure 11). Increasing temperature reduces desaturation and overall FA quantity, which indicates that heat interferes with either fatty acid synthesis and/or stimulates fatty acid breakdown. On average, the relative proportion of unsaturated fatty acids in dried fronds was higher than in hydrated fronds (Supplementary Table 1), which may protect the temperature sensitive photosynthetic apparatus (Gombos et al., 1994). Additionally, unsaturation declined in dried fronds at temperatures > 40 and > 35°C in hydrated fronds (Figure 11), suggesting that heat stress increases saturation of fatty acids. Saturated FAs reduce membrane fluidity and enhance integrity (Ahmad et al., 2013; Sakhno et al., 2014). Both parameters are important for surviving high temperatures (Murata and Los, 1997; Upchurch, 2008). Additionally, the presence of polyunsaturated fatty acid makes membranes susceptible to peroxidation (Liu and Huang, 2004), which corresponds to our observation of higher LOOH in heat-stressed, dry fronds compared with hydrated fronds (Figure 8). Although saturated fatty acids (16:0 and 18:0) made up a small fraction of the total fatty acid pool (Figure 11), their presence likely contributes to membrane stability and heat tolerance (Liu and Huang, 2004; Ahmad et al., 2013).

The decline of all fatty acids at elevated temperatures (Figure 11) links the fatty acid content to senescence (Koiwai et al., 1981; Yang and Ohlrogge, 2009). Even though senescence is not detectable in dried fronds, high amounts of lipid hydroperoxides (Figure 8) imply that dehydrated fronds are also affected by heat-induced membrane damage. The sensitivity of FA spectra to temperature and hydration indicates that FA profiling can be an effective stress indicator. Future studies will decipher how the FA profile changes with the extent and duration of stress, and during recovery. Dried material undergoes metabolic reactions of lipids (Golovina et al., 2010) and longevity has been attributed to “differences in the structure or mobility of molecules within the solidified cytoplasm” (Ballesteros et al., 2017). The remaining elasticity of dried Pleopeltis fronds suggests that they are below the glass transition threshold and therefore capable of low-level metabolism. The changing levels of FAs are indicative of ongoing metabolism in tissue with low water content (Figure 11). One could speculate that the stress response to drying includes enhanced lipid biosynthesis (Quartacci et al., 2002) to maintain membrane fluidity (Murata and Los, 1997; Ahmad et al., 2013) and that elevated temperatures enhance respiration such that beta oxidation of FAs leads to their lowered content. Future studies will analyze the time course of these rather drastic changes, and how the FA profile changes during recovery.

## Conclusion

The mechanism of Pleopeltis response to heat stress is complex. Our biochemical data indicate that > 40°C causes heat stress in Pleopeltis fronds. Despite obvious stress signals, dried fronds recovered after exposure up to 55°C while thermo-tolerance of hydrated fronds was limited to 40°C. The difference in heat tolerance between hydrated and dried fronds indicates that dehydration protects Pleopeltis from heat damage. Our data further illustrate the adaptability of this epiphytic fern to cope with environmental stress.

## Data Availability Statement

The raw data supporting the conclusions of this article will be made available by the authors, without undue reservation.

## Author Contributions

SJ and KH conceived the experiments, analyzed the data, and wrote the manuscript. SJ performed the research. Both authors contributed to the article and approved the submitted version.

## Conflict of Interest

The authors declare that the research was conducted in the absence of any commercial or financial relationships that could be construed as a potential conflict of interest.
