# Supplementary material for: Desiccation Mitigates Heat Stress in the Resurrection Fern, Pleopeltis polypodioides
Source: Front Plant Sci. 2020 Nov 30;11:597731. doi: 10.3389/fpls.2020.597731 (PMC7733933; doi:10.3389/fpls.2020.597731)
Supplement: Supplementary file 1 [file Table_1.DOCX]

Supplements:

**Table 1:** Percentage of unsaturated fatty acids in Pleopeltis fronds as a function of temperature and hydration.

| Temp | Submersed | Partly submersed | Dry |
| --- | --- | --- | --- |
| 25 | 67.6 ± 3.8 | 67.6 ± 3.8 | 80.1 ± 3.2 |
| 30 | 68.7 ± 3.8 | 63.0 ± 3.9 | 67.7 ± 3.1 |
| 35 | 65.6 ± 3.6 | 56.3 ± 4.0 | 67.1 ± 3.0 |
| 40 | 64.8 ± 3.7 | 55.5 ± 3.8 | 67.5 ± 2.7 |
| 45 | 64.0 ± 3.6 | 54.4 ± 3.9 | 66.3 ± 2.8 |
| 50 | 59.1 ± 4.0 | 50.8 ± 4.4 | 64.2 ± 3.0 |

Unsaturation percentage for each temperature treatment is calculated as the ratio of sum of un-saturated fatty acids to total fatty acids, i.e.[(18:1) + (18:2) +(18:3)] / [(16:0) + (18:0) + (18:1) + (18:2) + (18:3)] × 100.
